# The multifaceted role of lysine acetylation in cancer: prognostic biomarker and therapeutic target

**DOI:** 10.18632/oncotarget.10048

**Published:** 2016-06-14

**Authors:** Marta Di Martile, Donatella Del Bufalo, Daniela Trisciuoglio

**Affiliations:** ^1^ Preclinical Models and New Therapeutic Agents Unit, Research, Advanced Diagnostics and Technological Innovation Department, Regina Elena National Cancer Institute, Rome, Italy

**Keywords:** lysine acetylation, lysine acetyltransferases, KAT inhibitors, cancer

## Abstract

Lysine acetylation is a post-translational modification that regulates gene transcription by targeting histones as well as a variety of transcription factors in the nucleus. Recently, several reports have demonstrated that numerous cytosolic proteins are also acetylated and that this modification, affecting protein activity, localization and stability has profound consequences on their cellular functions. Interestingly, most non-histone proteins targeted by acetylation are relevant for tumorigenesis. In this review, we will analyze the functional implications of lysine acetylation in different cellular compartments, and will examine our current understanding of lysine acetyltransferases family, highlighting the biological role and prognostic value of these enzymes and their substrates in cancer. The latter part of the article will address challenges and current status of molecules targeting lysine acetyltransferase enzymes in cancer therapy.

## INTRODUCTION

## LYSINE ACETYLATION: LYSINE ACETYLTRANSFERASES (KATS) AND LYSINE DEACETYLASES (KDACS)

Acetylation of the ε-amino group of lysine residues has recently emerged as an important covalent post-translational modification (PTM) for regulating protein functions. Acetyltransferases catalyze the transfer of an acetyl group from acetyl-CoA to the terminal amine on the side chain of lysine residues. These enzymes are commonly called Histone acetyltransferases (HATs), because their best-known substrates are histone proteins (H2A, H2B, H3 and H4). However, the nomenclature is being changed to lysine acetyltransferases (KATs), reflecting their ability to acetylate lysine (K) on many proteins. Conversely, histone or lysine deacetylases (HDACs or KDACs), catalyze the inverse reaction by removing acetyl groups from proteins (Figure 1). The conversion of the positively charged lysine to acetyl-lysine, like the addition of negative phosphates to uncharged amino acids during phosphorylation, alters protein structure and interactions with other biomolecules.

**Figure 2:**
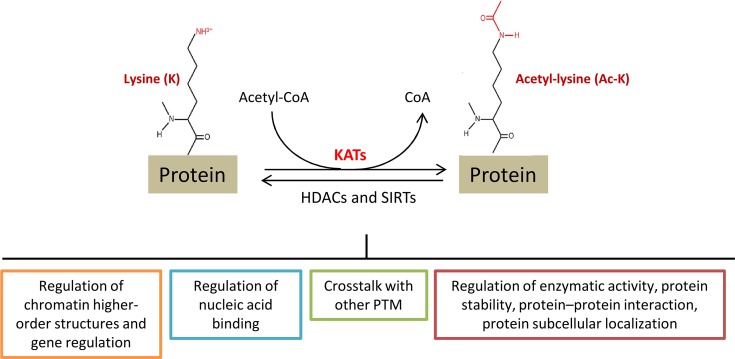
Lysine (K) acetylation is a reversible post-translational modification of proteins, including histones, transcription factors as well as metabolic enzymes and other nuclear and cytoplasmic proteins Proteins can be acetylated at lysine residues (Ac-K) by specific enzymes, called lysine acetyltransferases (KATs), or deacetylated by Zn^2+^-dependent histone deacetylases (HDACs) and NAD^+^-dependent sirtuin deacetylases (SIRTs). Acetylation levels are tightly regulated by these enzymes and can impact the biological function of different proteins, some of them are here reported. PTM: post-translational modifications.

Thus far, different KATs, localized both in the nucleus and cytoplasm, have been identified in human cells [1, 2]. They can be grouped into three major families: Gcn5-related N-acetyltransferase (GNAT), p300 and CREB-binding Protein (p300/CBP) and Moz, Ybf2/Sas3, Sas2, Tip60 (MYST). Apart from these, two other KAT families exist, which belong to transcription factor-related KATs and nuclear receptor family of KATs (Table 1). The 18 KDACs that have been identified in the human genome belong to two distinct families with different catalytic mechanisms: Zn^2+^-dependent histone deacetylases (HDAC1-11) and NAD^+^-dependent sirtuin deacetylases (SIRT1-7). Zn^2+^-dependent deacetylases are predominantly expressed both in the nucleus and cytoplasm, whereas sirtuins are present also in the mitochondria.

In the present review, we discuss the functional implications of lysine acetylation in different cellular compartments, the changes in lysine acetylation associated with cancer biology and the potential relevance of lysine acetylation as biomarkers for cancer prognosis. Finally, we discuss the achievements and drawbacks of drugs targeting the lysine acetylation, with particular regards to the field of anti-neoplastic drug development.

**Table 1:** Lysine acetyltransferases (KATs) family

New nomenclature	Former name in human	Cellular localization	Histone protein acetylated	Main non-histone protein acetylated
**P300/CBP family**				
KAT3				
KAT3A	CBP	Nucleus	H2A, H2B	NF-kappaB, c-myb, Foxo1
KAT3B	P300	Nucleus	H2A, H2B	NF-kappaB, c-myc, p53, STAT3, β-catenin, Foxo1, AR
**GNAT family**				
KAT1	HAT1	Nucleus	H3, H4, H2A	
KAT2			H3, H2B	
KAT2A	GCN5	Nucleus	H3, H4, H2A	CDC6, CDK9,cyclin D1, cyclin E1 and E2F1, HDM2, PTEN, c-myc
KAT2B	pCAF	Nucleus	H3	p53, CDK9, c-myc, Foxo1, AR
KAT9	ELP3		H4, H2A, H3	
ATAT-1	MEC-17	Cytosol		tubulin, cortactin
AT-1		ER		BACE-1, ATG9
AT-2		ER		BACE-1
**MYST family**				
KAT5	Tip60	Nucleus	H4, H2A	ATM, TRRAP, p53, E2F1, c-myc, DNMT1
KAT6				
KAT6A	MOZ	Nucleus	H3	
KAT6B	MORF	Nucleus		
KAT7	HBO1	Nucleus	H3, H4	
KAT8	MOF		H4	

## LYSINE ACETYLTRANSFERASES FAMILIES AND THEIR LINK TO CANCER

### p300/CBP family

The p300/CBP family is composed of two closely related transcriptional coactivators CBP (or KAT3A) and its paralog p300 (KAT3B). The human*CBP*locus resides in the chromosomal region 16p13.3 and shows homology to 22q13, where *p300* is located. They have also similar structures and share an overall 63% amino acid sequence identity and around 86% sequence identity at the KAT domain. CBP and p300 have interchangeable roles during embryonic development, and in many processes they govern cellular homeostasis. Both are transcriptional co-activators of various sequence-specific transcription factors that are involved in a wide array of cellular activities, such as DNA repair, cell growth, senescence, differentiation and apoptosis [3]. p300 is also involved in the regulation of expression and function of a large number of tumor-relevant proteins, including oncoproteins c-myc [4], androgen receptor (AR) [5], tumor suppressor proteins breast cancer gene-1 (BRCA1) [6] and p53 [7].

The importance of p300/CBP is underscored by the fact that genetic alterations, as well as their functional dysregulation, are strongly linked to cancer. Germline mutations of *CBP* were firstly reported in Rubinstein-Taybi Syndrome, an autosomal-dominant disease characterized by mental retardation, skeletal abnormalities and a high malignancy risk. Most of the described tumor-related mutations in *p300/CBP* result in truncation of the p300 protein. Mutations and/or deletions of *p300* and/or *CBP* genes have been also reported in several types of cancer, as lung, colon, breast and ovarian carcinomas [8–10], indicating a p300 role as tumor suppressor, and suggesting that it may play a role in the development of a subset of human cancers. In this context, loss of heterozygosity (LOH) at the *p300* locus has been observed in numerous cancers, including hepatocellular, colorectal, oral, breast, ovarian, gastric carcinomas and glioblastomas [11]. Consistently, several studies have also shown that loss of *p300* correlates with aggressive features and poor prognosis of hepatocellular carcinoma (HCC) [12, 13], breast cancer [14], cutaneous squamous cell carcinoma (SCC) [15] and nasopharyngeal carcinoma [16]. However, p300 is also found to be overexpressed in prostate cancer, where it regulates fatty acid synthase expression, lipid metabolism and prostate cancer growth [5, 17, 18]. *p300* and *CBP* genes are involved in various chromosomal translocation events during haematological malignancy and might contribute to aberrant growth control possibly through a gain of function mutation. For example, the chromosomal translocation events that affect *CBP* give rise to tumor-specific hybrid proteins [19, 20]. In particular, chromosome translocations targeting *CBP* have been found in acute myeloid leukemia (AML) and are associated with the development of this neoplasia following chemotherapy for other forms of cancer [21]. Recently, it was shown that the *CBP* gene is genetically altered in almost 15% of lung cancer cell lines and 5% of primary lung tumors. An interesting coexistence of *CBP* and *p53* mutations was also observed in lung cancer, suggesting that *CBP* gene alterations might contribute to lung carcinogenesis by distorting pathways other than those engaging p53 [8].

### GNAT super family

The GNAT super family includes about 12 proteins with diverse cellular functions and substrates, among them GCN5 (General Control Nonderepressible 5; KAT2A) and other proteins showing a sequence and structural similarity to GCN5, PCAF (p300/CBP Associated Factor; KAT2B), α-tubulin acetyltransferase 1 (ATAT1), the chromatin-assembly-related Hat1, the elongator complex subunit Elp3, the mediator complex subunit Nut1, and Hpa2. GNAT proteins share a domain composed of four conserved sequence motifs A-D, and unusually they also have bromodomain or chromodomain for binding acetylated or methylated lysine respectively [22].

The two main members of this family, GCN5 and PCAF are closely related proteins. The former has homologs in yeast and human, whereas the latter appears exclusively in higher eukariotes. In general, GNATs are involved in cellular growth, playing an important role in the regulation of cell cycle. For example, GCN5 specifically acetylates cell-division cycle-6 (CDC6) at three lysine residues flanking its cyclin-docking motif. This modification is crucial for the subsequent phosphorylation of the protein by cyclin A-cyclin-dependent kinase (CDKs) at a specific residue close to the acetylation site. GCN5-mediated acetylation and site-specific phosphorylation of CDC6 are both necessary for the relocalization of the protein to the cell cytoplasm in the S phase, as well as for the regulation of its stability [23]. Both GCN5 and PCAF, regulate cyclin-dependent kinase-9 (CDK9) function by specifically acetylating the catalytic core of the enzyme. This modification causes a profound inhibition of CDK9 catalytic and transcriptional activities and relocates the enzyme to the insoluble nuclear matrix compartment [24]. In non-small cell lung cancer (NSCLC), GCN5 enhances cell proliferation and G1/S transition by regulating the expression of cell cycle proteins like cyclin D1, E1 and E2F1 [25]. Under stress conditions, PCAF is required for stress-responsive histone H3 acetylation at the p21 promoter, p53-directed transcription of p21 and the resultant growth arrest [26]. Interestingly, it was also shown that PCAF possesses a ubiquitin ligase activity, that is a key determinant in controlling Hdm2 levels and, consequently, the stability and activity of p53 [27]. PCAF has been also reported to interact with the tumor suppressor PTEN and to promote its acetylation on K125 and K128 in response to growth factor stimulation [28]. Moreover, PCAF has also been shown to be a p53 target gene [29].

The involvement of GCN5, PCAF and other family members in processes that are closely linked to the hallmarks of cancer, including DNA damage repair, cell cycle regulation and post-translational regulation of both oncoproteins and tumor suppressors, suggests an important functions for these proteins in cancer progression.

*GCN5* is found to be overexpressed in human glioma and NSCLC tissues, where its expression positively correlates with proliferation of cell nuclear antigen (PCNA) and tumor size [25, 30], respectively. Recently, GCN5 expression was found upregulated in human colon cancer and regulated by c-myc and E2F1 transcription factors [31].

*PCAF* maps to the short arm of chromosome 3, which is frequently deleted in solid tumors such HCC, ovarian cancer, gastric cancer and esophageal SCC. Accordingly, *PCAF* has been found to be down-regulated in HCC tissues compared with the adjacent non-tumor tissues and significantly associated with malignant portal vein invasion and poor survival of HCC patients [32]. *PCAF* expression has also been reported to be downregulated in HCC, gastric cancer and ovarian cancer [33]. In esophageal SCC, loss of *PCAF* locus correlated with advanced tumor stage and metastasis. The expression of *PCAF* was also found to be downregulated in esophageal SCC cell lines and this downregulation was associated with DNA hypermethylation at the PCAF promoter [34].

ATAT1 is the main KAT responsible for α-tubulin acetylation at K40 in higher organisms. Recent structural analyses have provided elegant insights into how ATAT1 targets and acetylates K40 of α-tubulin [35–37]. Recent reports suggest that ATAT1 plays a prominent role in many cellular processes related to cancer dissemination, including cell adhesion, migration and invasion. In breast cancer cells, ATAT1 binds and regulates cortactin acetylation levels and colocalizes with cortactin at the adherent surface of the cells. ATAT1 is required for 2D migration and invasive capability of breast cancer cells in collagen matrix [38]. Ectopic ATAT1 expression in cultured cells also increases tubulin acetylation and enhances formation of microtentacles, which are membrane protrusions in detached breast cancer cells [39]. Notably, *ex vivo*cultured*ATAT1*−/−mouse embryonic fibroblasts displayed impaired cell adhesion and contact inhibition [40]. ATAT1 is also associated with pancreatic cancer-initiating cells [41]. A recent study on cancer cells showed that an efficient ATAT1 downregulation could impair actin architecture and induce mitotic catastrophe in cells through mechanisms partly independent of acetyl-tubulin [42]. Interestingly, in zebrafish ATAT1 appears to govern embryo development [43], conversely the viability and grossly normal development of *ATAT1*−/−embryos and mice indicate that ATAT1 is not essential for basic functions such as animal survival and development, but it may be important for more advanced functions, including learning and memory [44].

### MYST family

MYST family is named after its founding members MOZ, Ybf2/Sas3, Sas2, Tip60 in yeast. The family currently comprises five human KATs: Tip60 (HIV1 TAT interacting 60 kDa protein), MOF, MOZ (monocytic leukemic zinc-finger protein), MORF (MOZ related factor), HBO1 (histone acetyltransferase bound to origin recognition complex (ORC)).

MYST members are characterized by the presence of a conserved MYST domain containing an acetyl-CoA binding and a zinc finger motifs [45]. Many MYST also have other domains for recognizing other proteins and are involved in transcription control, as well as cell growth and survival [46]. Surprisingly, this protein family was poorly studied. However, an increasing number of key studies have recently shown that these chromatin modifiers are required for a diverse range of cellular processes, both in health and disease.

Tip60, a well-studied member of the MYST family, is implicated in multiple cellular pathways, such as transcription, DNA damage-induced checkpoint activation, and apoptosis. It is an important enzyme for repairing DNA and returning cellular function to normal through its regulation of ataxia telangiectasia mutant (ATM) protein kinase, which phosphorylates and therefore activates proteins involved in DNA repair. However, in order to be functional, ATM protein kinase must be acetylated by the Tip60 protein. Lack of Tip60 suppresses ATM protein kinase activity and reduces the ability of a cell to repair DNA [47]. In the DNA repair process, Tip60 serves as a cofactor for transformation/transcription domain associated protein (TRRAP), an adapter protein playing an important role in Double Strand Break repair and chromatin remodeling. TRRAP enhances DNA remodeling by binding to chromatin near broken double stranded DNA sequences, and Tip60 seems to aid this recognition [48]. Acetylation of p53 by Tip60 at K120 was also reported and it was demonstrated to be essential for p53 induction of cell death [49]. Moreover, Tip60 knockdown leads to cisplatin-sensitivity in lung cancer cell lines. During DNA damage response to cisplatin, Tip60 interacts with E2F1 and promotes the E2F1 acetylation necessary for its accumulation [50].

Given the roles of Tip60 in transcriptional activation and DNA repair, it is not surprising that this KAT has been linked to cancer. The human *Tip60* locus is frequently mutated in head and neck SCC, breast carcinoma and lymphomas. Nuclear Tip60 staining on tissue microarrays is lost in a variety of tumors, and most significantly in breast carcinomas [51]. Recent studies show down-regulation of Tip60 in breast cancer, and a correlation between low Tip60 levels and *p53* mutations in basal-like breast cancers, thus suggesting that Tip60 is a novel breast tumor suppressor gene whose loss results in genomic instability leading to cancer formation [52]. Tip60 can also function as a co-activator for a number of steroid hormone receptors including the AR, which is involved in the development and progression of prostate cancer. In line with this, Tip60 is functionally up-regulated in clinical prostate cancer specimens and its expression correlates with disease progression [53].

Human MOF is highly conserved from fly to human and shows the same substrate specificity [54]. It is responsible for the larger part of K16 acetylation at histone H4 in human cells, with obvious links to cancer [55, 56]. In fact, it has been well known that depletion of MOF can influence a wide range of intracellular biological functions, including chromatin stability, cell cycle, gene transcription, DNA damage repair and early embryonic development [54, 57]. MOF has also been shown to play a role in embryonic stem cell renewal. In particular, MOF is an integral component of the embryonic stem cell core transcriptional network and it primes genes for diverse developmental programs, thus playing a very important role in normal physiology and disease [58]. Except for the global reduction of histone H4 acetylated at K16, depletion of MOF in mammal cells can result in abnormal gene transcription, especially causing abnormal expression of certain tumor suppressors or oncogenes [57, 59]. For example, MOF is frequently downregulated in 41% of patients with primary breast carcinoma and in 79% of patients with medulloblastoma, and its protein expression is tightly correlated with acetylated H4 at K16 in all tested samples [60]. Notably, correlation of low expression of MOF with clinicopathological features of colorectal, gastric and renal cell carcinomas was reported, and in patients with colorectal cancer, the patterns of MOF expression were mainly associated with lymph node metastasis and tumor stage [61]. Loss of MOF levels is prognostic marker also in ovarian and hepatocellular carcinomas [62–64]. Conversely, the overexpression of MOF in NSCLC predicts poor prognosis of the disease [65].

Human MOZ and MORF form stable multisubunit complexes, which are responsible for acetylation of a substantial portion of histone H3. The KAT activity of MOZ/MORF complexes is required for normal developmental programs, including hematopoiesis and skeletogeneis, and for the regulation of various genes, especially the Hox family [46, 66–69]. Both *MOZ* and *MORF* are mutated in cancer and developmental disorders. Chromosomal aberrations involving *MOZ* and *MORF* may be a cause of AML. In AML, both *MOZ* and *MORF* fuse with multiple genes including *CBP* and *p300* [19, 20]. The resulting proteins possess 2 catalytic KAT domains, one from the MOZ/MORF fragment and another from the p300/CBP fragment, that trigger an aberrant histone acetylation and transcriptional activation associated with overexpression of oncogenes.

The inversion inv(8)(p11;q13) generates the MOZ-TIF2 fusion protein, that is associated with AML. MOZ-TIF2-induced transformation requires the MOZ nucleosome binding motif and TIF2-mediated recruitment of CBP [70]. In a recent report, mutant p53 has been shown to upregulate chromatin regulatory genes expression, including MOZ, leading to alteration in global chromatin modification. This may contribute to the gain of function of mutant p53. Interestingly, the Cancer Genome Atlas shows specific upregulation of MOZ in p53 gain of function patient-derived tumors, but not in *wild-type* p53 or p53 null tumors [71]. Recently, the presence of homozygous deletions of MORF, and the loss of the corresponding transcript, were observed in small cell lung cancer cell lines and primary tumors. Notably, *in vitro* and *in vivo* evidence demonstrated that the depletion of MORF expression enhances cancer growth, whilst its restoration induces tumor suppressor-like features [72].

A fifth human MYST protein is HBO1, which was discovered in a two-hybrid screen on the basis of its interaction with the ORC1 subunit of the origin recognition complex ORC [73]. HBO1 appears to play an essential role in DNA replication, so it is reasonable to speculate that defects in its function should have profound consequences to the cell and lead to oncogenesis. Nevertheless, little it is known on the role of HBO1 in cancer [46].

## FUNCTIONAL IMPLICATIONS OF LYSINE ACETYLATION IN DIFFERENT CELLULAR COMPARTMENTS: BIOLOGICAL ROLE AND SIGNIFICANCE IN CANCER

In the last years, developments of proteomic innovations have propelled acetylation research forwards. In 2009, by using high-resolution mass spectrometry, Mann and colleagues looked in deep to the cellular acetylome [74]. They identified about 3,600 lysine acetylation sites in 1,750 proteins, and observed the presence of lysine acetylation on proteins localized in different cellular compartments. Nowadays, it is well clear that lysine acetylation is not exclusively limited to nucleus (Figure 2), but may also occur in other cellular compartments, suggesting that acetylation may also regulate cellular processes not directly related to transcriptional activation (Figure 3).

**Figure 2:**
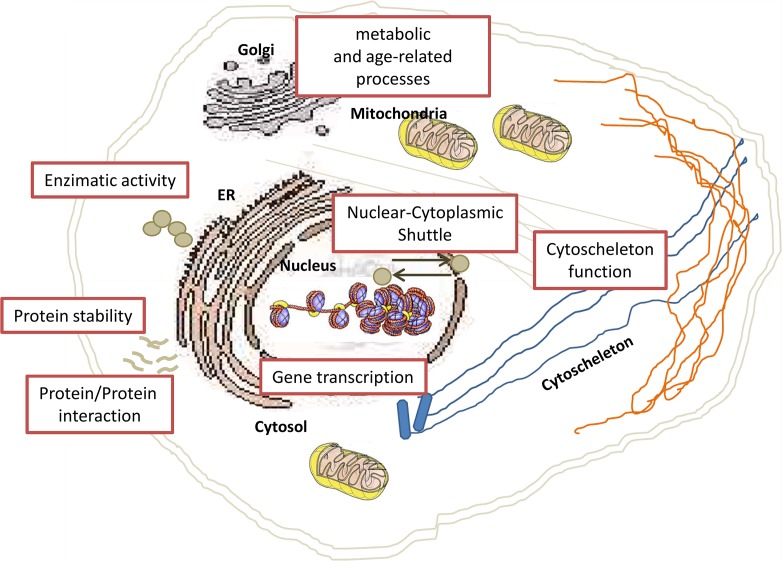
Schematic overview of nuclear, cytoplasmic and organelle-specific mechanisms regulated by acetylation Lysine acetylation is well known to play a key role in regulating gene transcription and other DNA-dependent nuclear processes. Proteomics studies have identified many possible substrates of lysine acetylation and a large fraction of them resides in the cytoplasmic compartment, implicating their involvement in regulating important cellular pathways.

**Figure 3:**
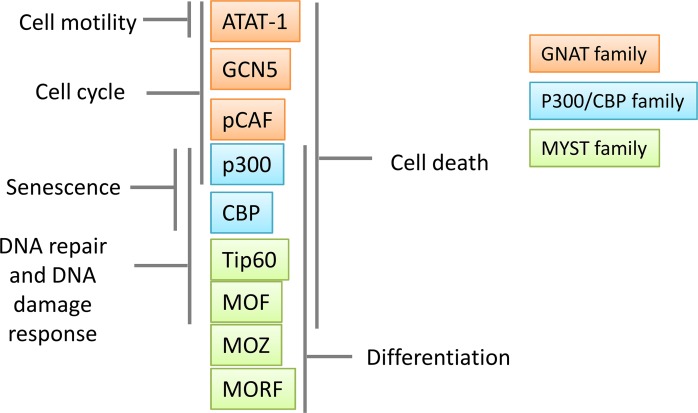
Role of main and well-studied lysine acetyltransferases (KATs) in cancer biology KATs have an important role in transcription regulation and they participate in the expression of malignant phenotypes in cancer cells.

## NUCLEUS: ACETYLATION OF HISTONES AND TRANSCRIPTION-RELATED FACTORS

KATs are intimately involved in transcriptional activation. By modifying chromatin proteins and transcription-related factors, these enzymes are believed to regulate the transcription of many genes involved in cancer. In particular, chromatin architecture is strongly regulated by PTM of the N-terminal tails of the histone proteins [75]. Thus, acetylated histones represent a type of epigenetic markers within chromatin. In general, the presence of acetylated lysine in histone tails is associated with an open chromatin configuration that provides accessibility for specific transcription factors and the general transcription machinery. HDACs remove the acetyl groups, allowing formation of compacted chromatin that is associated with transcriptional gene silencing. Hence, the levels of histone acetylation play a crucial role in chromatin remodeling and in the regulation of gene transcription. Notably, histone acetylation also results in the recruitment of transcription and chromatin remodeling factors. Such factors are typically recruited through a bromodomain (BRD), functioning as acetyl-lysine binding domains.

Proteins containing one or more BRDs belong to BET (Bromodomain and extraterminal domain) family, an evolutionarily conserved family of proteins associated with chromatin and present in nearly all nuclear KATs. These proteins perform transcription regulatory functions under normal conditions, while in cancer they regulate transcription of several oncogenes, such as c-Myc and Bcl-2 [76]. Notably, different PTMs may reside on the same histone molecule. For example, PTMs neighboring acetylated lysines can strongly alter the affinity of BRD—and likewise, the enzymatic of KATs and HDACs may be modulated by close-by PTMs [77]. Recently in an elegant paper Feller and coworkers have applied a mass spectrometry-based strategy to generate a comprehensive catalog of combinatorial histone acetylation and other PTM motifs in Drosophila cells. In particular, they have described the histone acetylation changes in response to ablation of known or suspected KATs and HDACs [78]. Surprisingly, they have found that depletion of single KAT activities leads to complex alterations of the epigenome including, the reduction of primary substrates, the global redistribution of acetyl groups to secondary sites, and changes to methylation of histones.

Many studies have suggested alterations in histone acetylation as potential diagnostic or prognostic biomarkers in human diseases such as cancer [75]. Recently, there has been a significant growth in our knowledge about the involvement of aberrant patterns of histone modifications in cancer development. Analysis of global histone modification in a large cohort of solid cancer has instead revealed differential levels of bulk histone acetylation across tumors, and the occurrence of some of these histone marks correlates with tumor morphology and biological subtype. In general, tumors with adverse traditional prognostic or phenotypic characteristics were found to have reduced levels of detectable H3 and H4 acetylation. Loss of selected histone acetylation and methylation marks has been shown to predict patient outcome in human carcinoma [79]. For example, acetylation of H3 at K56 is often increased in multiple types of cancer and undifferentiated cells [80]. Barlesi and collaborators showed that acetylation of histone H2A at K5 and H3 at K9, as well as histone H3 di-methylation at K4, influenced overall and disease-free survival of NSCLC patients who had undergone resection, suggesting the role of epigenetic modifications in lung carcinogenesis [81]. In another study, cancer cells compared with normal lung displayed an aberrant pattern of histone H4 modifications with hyperacetylation at K5 and K8, hypoacetylation at K12 and K16, and loss of K20 trimethylation [82]. A recent study on immunostaining analyzed 408 NSCLC tissues, showing that global histone H3 and H4 modification patterns are potential markers of tumor recurrence and disease-free survival [83]. Breast cancer patients analysis reveals that the loss of acetylated H4 at K16 may serve as an early sign of cancer, and low levels of H3 acetylated at K9 and K14 and H4 at K12 are prognostic of poor outcomes [84]. Independent studies have demonstrated the prognostic significance of histone acetylation in esophageal SCC [85, 86]. In particular, abnormal levels of H3 and H4 acetylation correlate with the severity and histological differentiation of this tumor histotype.

In general, acetylation can lead to increase in DNA binding affinity of transcription factors such as c-myb, STAT3, E2F1 [87, 88], this in turn could lead to increase in transactivation and gene expression by these proteins. Stat3 is acetylated by p300 and the major Stat3 acetylation site is located at K685. The acetylation on Stat3 enhances its DNA binding and transactivation activities, and increases its nuclear localization [88]. In the case of c-myb, acetylation by CBP increases the transactivating capacity of c-myb by enhancing its association with CBP [87]. pCAF acetylates one of the cell-cycle regulator E2F family member, E2F1, at the DNA binding domain, which increases DNA binding ability of the protein and thus E2F mediated transcriptional activation. Other acetylases (GCN5, CBP and p300) can also acetylate E2F1, but much less efficiently [89]. Conversely, in the case of FoxO1, three acetylation sites (K242, K245, and K262) located in the protein domain that directly participates in DNA recognition and/or stabilization of the FoxO-DNA complex, were identified and their acetylation attenuates FoxO1 transcriptional activity. In fact, the positive charge of these lysines in FoxO1 contributes to its DNA-binding, and acetylation at these residues by CBP attenuates its ability to bind cognate DNA sequence [90].

Acetylation of transcription factors can alter their activity, but this is dependent on the functional domains that are acetylated. For example, the tumor suppressor p53 contains a lysine-rich basic domain near its C-terminus. Six different lysine residues, spanning sites 370-386 on human p53, can be modified by acetylation and other PTM that facilitates p53 activation. However, acetylation may serve multiple roles for p53, including stabilizing the protein, altering association with other proteins or other p53 monomers, enhancing its binding to DNA, and regulating transcription [91].

## CYTOSOL: ACETYLATION OF CYTOSKELETON-ASSOCIATED PROTEINS, NUCLEAR-CYTOPLASMIC SHUTTLE, AND OTHER CELLULAR FACTORS

Lysine acetylation plays a crucial role in the regulation of cytoskeleton-associated proteins including actin and tubulin. There are three major isoforms of actin. β and γ actins form the stress fibers and are important for cell shape and cell movement. α-actin forms the core of the thin filament of the sarcomere where it interacts with a variety of proteins to produce the force for muscle contraction. Using proteomic approaches it has been shown that all three actin isoforms can be acetylated [74, 92] and acetylation of K61 in γ-actin may result in stabilization of actin stress fibers [92]. Several regulatory proteins of the actin cytoskeleton are also modified by acetylation. In particular, cortactin is regulated through acetylation by histone acetyltransferase p300, and deacetylation by HDAC6 and SIRT1. Acetylation abrogates binding of cortactin to filamentous actin and leads to diminished actin dynamics and altered cell motility [93, 94].

Like actin, the cytoskeletal protein α-tubulin is also acetylated, with the K40 residue on α-tubulin identified as the acetylation site. Acetylation of α-subunit tubulin occurs in a context of tubulin assembly, which is conserved among species. In mammals, the responsible enzymes have been identified and characterized as ATAT1 and HDAC6 [95]. Using a proteomic approach for acetylated proteins, several other acetylated residues in different tubulin isoforms (α as well as β subunits) were detected, but their role and significance have still to be elucidated [74]. There is growing evidence that acetylation of tubulin globally impacts on cellular functions and several reviews have recently been devoted to that subject [95, 96]. Briefly, studies have shown that tubulin acetylation contributes to microtubule stabilization and allows for more efficient bundling of acetylation-stabilized microtubules. It is also implicated in other cell functions, including intracellular endoplasmic reticulum (ER) localization and ER-mitochondria interactions [97]. Moreover, elevated tubulin acetylation promotes adhesion and invasion of breast cancer cells, and a relationship between high level of α-tubulin acetylation and metastatic behaviour of basal-like breast cancers, has also been recently reported [39]. High level of acetylated tubulin have been also found correlated with a higher tumor grade in SCC of the head and neck [98], and its expression has been suggested as a prognostic marker in epithelial malignancies and as a marker for sensitivity to chemotherapy [99].

Subcellular distribution of many proteins can be also influenced by acetylation. Interestingly, for some proteins, acetylation favors localization to the cytoplasm, whereas for others, acetylation enhances the retention of proteins in the nucleus. For example, c-Abl acetylation on K730 leads it from nuclear to cytoplasmic delocalization and promotes myogenic differentiation [100]. Similarly, an increased acetylation on β-catenin at K49 resulted in increased membrane localization in human induced pluripotent stem cells derived from neuronal cells [101]. Conversely, acetylated form of eIF5A was primarily enriched in the nucleus, whereas unacetylated eIF5A was primarily cytoplasmic; this distinction was even more pronounced in the presence of HDAC inhibitors [102]. Acetylation of one nuclear localization signal sequence of Net1A, a Rho guanine-nucleotide-exchange factor, regulates its subcellular localization to impact RhoA activity and actin cytoskeletal organization [103].

In some cases, acetylation also competes with other modifications [104]. For some transcription factors such as p53, FoxO, and c-myc, it has been observed that protein stability can be increased by acetylation, through blocking ubiquitination of the same residues, which will target the protein for proteasomal degradation. Acetylation and ubiquitination were found to modify the same residues at the C-terminal domain of p53 (K residues number, 370, 372, 373, 381, 382). Thus, acetylation blocks the export and degradation of p53 preventing ubiquitination [91]. Ubiquitination and acetylation can occur on the same lysine residues as well on FoxO. Using acetylation-defective and acetylation-mimicking mutants, Kitamura et al. have found that these mutual effects play an important role in the FoxO1-dependent oxidative damage response in pancreatic β cells [105]. Also acetylation of c-myc by either GCN5/PCAF or Tip60 results in a dramatic increase in protein stability [106]. Alternatively, acetylation can also decrease protein stability, for example DNA methyltransferase 1 (DNMT1), the primary enzyme that maintains DNA methylation, is destabilized by Tip60 acetylation, which targets DNMT1 for proteasomal degradation [107].

Acetylation can also alter the enzymatic activity of certain enzymes or their ability to interact with other proteins. Lu and coworkers have demonstrated that MOF not only acetylates histone H4 at K16, but it also acetylates itself to regulate its recruitment and activities on the chromatin [108]. ATM kinase activity is tightly regulated by Tip60-dependent acetylation at K3016, which is located in the highly conserved C-terminal FAT domain adjacent to the kinase domain. Mutation of K3016 does not affect unstimulated ATM kinase activity but inhibits the conversion of inactive ATM dimers to active ATM monomers, and prevents the ATM-dependent phosphorylation of the p53 and CHK2 proteins [109].

## FUNCTIONAL IMPLICATIONS OF ORGANELLE-SPECIFIC ACETYLATION

For a long time, lysine acetylation was thought to be limited to the cytosol and nucleus, where both the donor of the acetyl group and KAT were available. On the contrary, over the last few years it has been proved that a large number of organelle-resident and/or organelle-transiting proteins undergo lysine acetylation in the lumen of the organelle, as mitochondrial and ER.

In 2006, an extensive proteomic survey of cellular proteins have revealed that a large number of mitochondrial proteins are subject to reversible lysine acetylation [92]. In this proteomic analysis of the acetylated peptides obtained from purified mouse liver mitochondria, 277 lysine acetylation sites were identified in 133 mitochondrial proteins. Thus revealing that at least 20% of all mitochondrial proteins are lysine acetylated and suggesting that lysine acetylation is an abundant PTM in the mitochondrion. A more recent proteomic analysis of lysine-acetylated mitochondrial proteins has shown that as much as 50% of all mitochondrial proteins are acetylated, and most of them are involved in energy metabolism. These include proteins involved in the tricarboxylic acid (TCA) cycle, oxidative phosphorylation, β-oxidation of lipids, amino acid metabolism, carbohydrate metabolism, nucleotide metabolism, and the urea cycle [110, 111]. It is well known that the three deacetylases (SIRT3, SIRT4, and SIRT5) located in the mitochondria are poised to mediate mitochondrial protein acetylation levels. Among them SIRT3 is localized in the mitochondrial matrix and its expression is selectively activated during fasting and calorie restriction. Activated SIRT3 deacetylates several key metabolic enzymes (e.g. acetyl-coenzyme A synthetase, long-chain acyl-coenzyme A (acyl-CoA) dehydrogenase (LCAD), and 3-hydroxy-3-methylglutaryl CoA synthase 2) and enhances their enzymatic activity [112]. Interestingly, mitochondrial acetyltransferases (MATs) have not been identified, raising the question as to how mitochondrial proteins become acetylated. One explanation is that high acetyl-CoA levels in the mitochondria could facilitate a similar non-enzymatic acetylation mechanism. Alternatively, MATs could mediate the acetylation reaction or mitochondrial proteins could be acetylated by a novel mitochondrial enzyme, not yet recognizable as a KATs. A multitude of mitochondrial proteins are now known to be acetylated. Some of them are modified in a reversible and nutrient-responsive manner. Accordingly, alterations in mitochondrial acetylation states, and, hence, alterations in carbon substrate utilization, may contribute to the unusual preference for aerobic glycolysis and glutaminolysis often observed in numerous forms of cancer [113].

In 2007, Costantini et al. reported transient dual enzymatic machinery acting both in the ER and Golgi apparatus. In particular, they have reported that membrane protein β-site APP cleaving enzyme 1 (BACE1), an aspartic-acid protease that processes amyloid precursor protein involved in the pathogenesis of Alzheimer's disease, is acetylated and deacetylated in seven different residues both in the ER and Golgi apparatus, respectively [114]. Subsequent proteomic studies have assessed the ER acetylome, and predicted wide-ranging biological implications of this pathway [115]. The list of ER-resident proteins includes chaperones and enzymes involved with PTM and folding. After that, many membrane and secreted proteins were reported to undergo transient lysine acetylation in the ER lumen [115–117]. This implies not only the presence of KATs in this cellular compartment but also an ER import system for acetyl-CoA. To date, ER acetylation has been found to be catalyzed by two different ER-based KATs, which were named AT-1 (also known as camello-like 2 and N-acetyltransferase 8B) and AT-2 (also known as camello-like 1 and N-acetyltransferase 8), both members of the camello family, which belongs to the GNAT superfamily [118]. Recently, AT-1 and consequently ER acetylation were genetically modified *in vivo* [119]. Importantly, the overexpression of AT-1 per se does not cause acetylation of proteins that are normally non-acetylated [117]. Targeting AT-1 function in mice leads to the appearance of neurodegenerative features, inflammation and cancer [119]. In conclusion, what was once a cytosolic and nuclear event now appears to be an essential component of mitochondria and ER functions as well.

## SMALL MOLECULE MODULATORS OF KATS AS CANCER THERAPEUTICS

The discovery of protein acetylation has led to the identification of many novel epigenetic drug targets. The first Food and Drug Administration-approved acetylation-modifying agent was the HDAC inhibitor vorinostat (also known as suberanilohydroxamic acid (SAHA)), initially identified as an agent inducing the differentiation of tumor cells *in vitro*. To date, other 3 HDACi (romidepsin, belinostat, and panobinostat) have been approved for cancer treatment and many other HDACi are currently used in clinical trials [120]. By contrast, the area of identifying KAT inhibitors (KATi) is not as well explored as HDACi (Table 2).

**Table 2:** Involvement of Lysine acetyltransferases (KATs) in cancer

KATs	Cancer Type	Alteration	Oncogene or tumor suppressor	Ref.
**p300/CBP**	Lung, colon, breast and ovarian cancers	mutation	tumor suppressor	[8–10]
	Hepato, colorectal, oral, breast, ovarian, gastric cancers and glioblastomas	loss of heterozygosity or deletion	tumor suppressor	[12, 13]
	Prostate cancers	overexpression	oncogene	[5, 17, 18]
	Haematological malignancies	chromosome translocations	oncogene	[19, 20]
**GCN5**	Glioma, colon and lung cancers	overexpression	oncogene	[25, 30]
**PCAF**	Hepato, ovarian, gastric and esophageal cancers	deletion	tumor suppressor	[33]
**Tip60**	Head and neck, breast cancers and lymphomas	mutation	tumor suppressor	[51]
	Prostate cancer	overexpression	oncogene	[53]
**MOF**	Breast, renal cell colorectal, gastric, ovarian, and hepato cancers, medulloblastoma	deletion	tumor suppressor	[60]
	Lung cancer	overexpression	oncogene	[65]
**MOZ/MORF**	Haematological malignancy	chromosome translocations	oncogene	[19, 20]
	Acute leukemia	chromosome translocations	oncogene	[70]
**MORF**	Lung cancer	deletion	tumor suppressor	[72]

The first KATi specific for p300 (Lys-CoA) and for PCAF (H3-CoA-20) were reported in 2000 [121]. The major drawback of these compounds is that they are cell impermeable, thus greatly restricting their utility. In 2005, Zheng and collaborators synthesized the first potent and selective KATi which is effective in live cells, by linking Lys-CoA and H3-CoA-20 to a cell permeabilizing ‘tat’ peptide [122].

In addition to bi-substrate analogues, several natural products have been reported as KATi. Curcumin, a polyphenol responsible for the yellow color of the spice turmeric, is the only KATi in clinical trials (https://clinicaltrials.gov/) and exhibits great promise as a therapeutic agent for its anti-inflammatory and antitumor properties. It possesses KAT inhibitory activity with specificity for the p300/CBP. In cells, curcumin promotes proteasome-dependent degradation of p300 and the closely related CBP protein without affecting PCAF or GCN5. Moreover, curcumin inhibits the KAT activity of purified p300 as assessed using histone H3 substrate [123]. Interestingly, it could also inhibit the acetylation of p53 [124]. The anticancer potential of curcumin stems from its ability to suppress cell proliferation of a wide variety of tumor cells. *In vitro* studies have demonstrated that curcumin is an efficient inducer of apoptosis and some degree of selectivity for cancer cells has been observed. Curcumin may also alter the effectiveness of radiotherapy and chemotherapy [125]. To date, about 57 clinical studies are ongoing with curcumin and its different formulations alone or in combination with conventional chemotherapeutic agents.

Anacardic acid, isolated from cashew nut shell liquid, has been identified as a potent inhibitor of p300 and PCAF, Tip60 KAT activity *in vitro* [126]. It has been shown that anacardic acid blocks Tip60-dependent activation of ATM and DNA-PK protein kinases by DNA damage *in vivo* [127]. Recently it was also found to inhibit MOF. Based on this evidence, Wapenaar and coworkers have designed a small collection of anacardic acid derived inhibitors and calculated their respective binding constants for MOF. This study will be the starting point for developing of selectively MOF inhibitors, which will ultimately enable the exploitation of this KAT as a novel drug target in disease [128].

Starting from the leading natural product KATi anacardic acid, a series of analogues was synthesized and investigated for KAT inhibitory properties and effects on cancer cell growth. The compounds have an improved inhibitory potency for PCAF/p300 KAT activity, and there is a clear correlation between their inhibitory potency and cytotoxicity toward a broad panel of cancer cells [129, 130]. MG153, a small molecule derived from anacardic acid, is a potent inhibitor of p300/PCAF, that decreases cell proliferation and induces apoptosis and resistance to DNA damage in BCR-ABL-expressing cells [131]. Interestingly, Pentadecylidenemalonate 1b, another analogue of anacardic acid, was identified as the first mixed activator/inhibitor of KATs. It potentiates PCAF KAT activity while inhibiting those of p300/CBP and recombinant CBP [132].

Garcinol is a polyisoprenylated benzophenone derivative isolated from *Garcinia indica*, and it is a potent inhibitor of PCAF and p300 enzymes. It was found to induce apoptosis and alter global gene expression in cancer cells. However, cells were found to be poorly permeable to this compound, thus limiting its practical application [124].

Epigallocatechin-3-gallate (EGCG) is the most abundant catechin in tea, and it has been found as a KATi in natural compound screening. EGCG can block p300-mediated acetylation of p65, impairing its translocation to the nucleus, thus inhibiting NF-κB activity and decreasing NF-κB target genes expression. EGCG impairs B cell transformation by Epstein-Barr Virus (EBV) and completely blocks EBV infection-induced cytokine expression Thus, EGCG could represent a potential therapy for B cell malignancies [133]. Plumbagin (RTK1), is a natural compound isolated from *Plumbago rosea* root extract, which inhibits p300 KAT activity potently *in vitro* and *in vivo*. Interestingly, RTK1 specifically inhibits the p300-mediated acetylation of p53. RTK1 also induces a distinct modification profile, involving transcriptional activation marks like histone H3 trimethylated at K4 and phosphorylated at S10 in the context of histone acetylation brought about by KAT3B/ p300 [134–136].

Recently, oridonin, a natural diterpenoid isolated from the Chinese medicinal herb *Rabdosia rubescens* was found to possess acetyltransferase-inhibitory effects on multiple KAT including p300, GCN5, Tip60, and PCAF. In gastric cancer cells, oridonin treatment inhibited cell proliferation and induced cell death by engaging the mitochondrial pathway of apoptosis, at least partially through p53-and caspase-3-mediated mechanisms [137].

In silico docking of commercial small molecule library has identified C646 as a competitive inhibitor of p300. It isa cell-permeable pyrazolone-containing small molecule with potent p300/CBP inhibitory activity with an IC50 value in the nanomolar range [138]. To date, C646 is the best characterized p300 KATi compound, which suppresses histone acetylation in human cancer cells *in vitro*. A recent study has been proposed C646 as potential candidate for treating AML1-ETO (AE) fusion protein-positive AML. C646 inhibits cellular proliferation, reduces colony formation, evokes partial cell cycle arrest in G1 phase, and induces apoptosis in AE-positive AML cell lines and primary blasts isolated from leukemic mice and AML patients. Notably, AE-positive AML cells are more sensitive to lower C646 doses than AE-negative ones. Finally C646 induces growth inhibition on AE-positive AML cells, and this event is associated with reduced global histone H3 acetylation and declined c-kit and bcl-2 levels [139]. C646 antitumor activity has been also show in different model of solid tumors. In prostate cancer cell lines, C646-mediated inhibition of p300 leads to induction of caspase-dependent apoptosis in several androgen-dependent and castration-resistant prostate cancer cells [140]. In NSCLC, Oike et al. have reported that C646 sensitizes cancer cells to radiotherapy, and enhances mitotic catastrophe induced by radiation [141]. C646 also inhibits the growth and promotes chemo-responsiveness of human melanoma cells. Mechanistically, p300 transcriptome has identified functional roles of p300 in promoting cell cycle progression, chromatin assembly, and activation of DNA repair pathways through direct transcriptional regulatory mechanisms [142]. Recently, a functional screening of synthetic-lethal genes in CBP-deficient cancers has proposed p300 inhibition as a promising therapeutic target for treatment of CBP-deficient cancers. In fact, ablation of p300 in CBP-knockout and -deficient cancer cells induces G1/S cell-cycle arrest, followed by apoptosis, and C646 specifically suppresses the growth of CBP-deficient lung and hematopoietic cancer cells *in vitro* and *in vivo* [143]. However, a recent chemical proteomic study has revealed that thiol reactivity of C646 may limit its ability to antagonize acetylation in cells [144]. In another recent study, a mass spectrometry analysis has shown a slight increase in acetylation of histone H3 in cells treated with high dose of C646. Indeed, they have also found an inhibition of HDACs at high concentrations, thus discovering a lack of selectivity of C646 at higher concentrations that needs to be taken into account for further development of C646 applications [145].

Recently, a novel cell-permeable p300/CBP-selective inhibitor, HATi II, has been described. HATi II inhibited the proliferation of a panel of glioma cells in a dose-dependent manner, by inducing cell cycle arrest at the G2/M phase, and apoptosis. In addition, microarray analysis and quantitative real-time PCR indicated that HATi II activates the p53 signaling pathway in glioma cells [146].

Small-molecule inhibitors of p300, such as L002 and isothiazolones, have been also identified by high-throughput screening as potent anticancer agents. L002 and its analogs have been identified from a library of 622,079 compounds. They inhibit the activity of p300 and related acetyltransferases (PCAF, and GCN5), but do not affect the activity of other KATs or HDACs or histone methyltransferases. Notably, *in vitro* leukemia, lymphoma and breast cancer cell lines are extremely sensitive to L002, whereas it potently suppresses tumor growth and histone acetylation of breast cancer xenografts [147].

Isothiazolones are antagonists of both p300 and PCAF [148]. Recently, two selected pyridoisothiazolone compound PU139, a pan-KATi, and PU141, a KAT3 selective KATi, were analyzed for their KAT inhibitory profile and for their anti-proliferative properties on a panel of human cancer cell lines. Interestingly, PU139 triggers caspase-independent cell death in cell culture, whereas both inhibitors block growth of neuroblastoma xenografts. Moreover, PU139 was shown to synergize with doxorubicin*in vivo*, and to reduce histone lysine acetylation*in vivo*at concentrations that block neoplastic xenograft growth [149]. Among isothiozolone-based inhibitors, 5-chloroisothiazolone has been identified as a PCAF specific inhibitor [150]. The thiazole-based synthetic compound, BF1 (1-(4-(4- chlorophenyl) thiazol-2-yl)-2-(propan-2-ylidene) hydrazine) can inhibit p300 and GCN5 *in vitro*, and when tested in both neuroblastoma and glioblastoma cell lines, it shows a global reduction in histone H3 and specific acetylation at K18 [151].

A series of cycloalkylidene-(4-phenylthiazol-yl)-hydrazone (CPTH) derivatives have been recently synthesized. The lead compound CPTH2 has been discovered through a phenotypic screening in yeast, then validated *in vitro* as a KATi. *In vivo*, it also decreases acetylation of bulk histone H3 at the specific H3 acetylated at K14 [152]. Among the first series of CPTH derivatives, our group has found that the compound named CPTH6 shows a time-dependent inhibition of KAT activity and acetylation of H3 and H4 histones in both AML and several solid tumors cell lines. We have also demonstrated that CPTH6 significantly reduces cell proliferation and induces cell cycle perturbation, apoptosis and monocytic-differentiation in U937 AML cells [153]. Interestingly, in a panel of cancer cell lines CPTH6 and analogs are able to modulate the autophagic flux inhibiting autophagosome maturation. Notably, CPTH6 treatment decreases α-tubulin acetylation and fails to increase autophagic markers in cells in which acetyltransferase ATAT1 expression was silenced, indicating a possible role of α-tubulin acetylation in CPTH6-induced alteration in autophagy [154]. More recently, the efficacy of CPTH6 has been tested on a panel of Lung Cancer Stem-like Cells (LCSC) derived from NSCLC patients. Notably, LCSCs exhibit greater growth inhibition than established NSCLC cells. Growth inhibitory effect of CPTH6 in LCSC lines is mainly due to apoptosis induction. Moreover, differentiated progeny of LCSC lines is more resistant to CPTH6 in terms of loss of cell viability and reduction of protein acetylation, when compared to their undifferentiated counterparts. Interestingly, CPTH6 is able to inhibit the growth of LCSC-derived xenografts and to reduce cancer stem cell content in treated tumors [155].

With the aim of identifying KATi more potent than the lead compounds CPTH2 and CPTH6, Carradori and collaborators have synthesized several new (thiazol-2-yl)hydrazones including some related thiazolidines and pyrimidin-4(3H)-ones, and tested the whole library against human p300 and PCAF KAT enzymes. Five compounds are more efficient than CPTH2 and CPTH6 in inhibiting the p300 enzyme, and when tested in human leukemia cells, they are more potent than CPTH6 in inducing apoptosis and cytodifferentiation [156].

Beyond anacardic acid and garcinol, only a small number of Tip60 inhibitors have been reported. Other Tip60 inhibitors include 6-alkylsalicylates [157], NU9056 an isothiazole-based KATi, which is effective against prostate cancer cells [158], Pentamidine a drug used for treating parasitic protozoan [159], and some small inhibitors reported by Wu and co-workers [160]. Recently a high-throughput screening has led to the identification of TH1834 as Tip60-selective inhibitor by using computational tools to design drugs based on the binding pocket of Tip60 TH1834 inhibits Tip60 *in vitro* and can sensitize breast cancer cells to ionizing radiation [161].

**Table:** Lysine acetyltransferase inhibitors (KATi) in cancer therapy

Category	Drug	Targeted KATs	Mechanism and main cellular effect	Ref.
Natural Compound				
	Curcumin	CBP, p300, PCAF	Promotes CBP/p300 degradation and inhibits KAT activity. Inhibits cell proliferation of a wide variety of tumor cells, and sensitizes to radiotherapy and chemotherapy. Affects various signaling pathways in cancer cells independently by its KAT inhibitory activity	[123,126]
	Anacardic acid (AA)	Tip60	Blocks the Tip60-dependent activation of ATM by DNA damage. Sensitizes human tumor cells to the cytotoxic effects of ionizing radiation	[127]
	Plumbagin (RTK1)	p300	Inhibits Ac-CoA and histone binding. Induces cell death and affects various signaling pathways in cancer cells	[134–136]
	Garcinol	PCAF, p300	Inhibits Ac-CoA and histone binding. Induces apoptosis and alters global gene expression in cancer cells	[124]
	Epigallocatechin- 3-gallate (EPGG)	p300	Blocks p300-mediated acetylation of p65, impairing its translocation to the nucleus, and inhibiting NF-κB activity and target genes expression	[133]
	Oridonin	p300, GCN5, Tip60, PCAF	Inhibits cell proliferation and induces apoptosis	[137]
Natural Compound analogs				
AA derivatives	1° series anacardic acid derivatives	p300, PCAF	Are cytotoxic toward a broad panel of cancer cells	[129,130]
	6-Alkylsalicylates	Tip60	Are competitive with Ac-CoA and non-competitive with the histone substrate	[157]
	Pentadecylidenemalonate 1b	PCAF, p300/CBP	Activates PCAF and inhibits p300/CBP, and induces apoptosis in cancer cells	[132]
	MG153	PCAF	induces apoptosis and resistance to DNA damage in BCR-ABL-expressing cells	[131]
Bisubstrate inhibitor				
	Lys-CoA	p300	Inhibits Ac-CoA and substrate binding	[122]
	H3-CoA-20	PCAF	Inhibits Ac-CoA and substrate binding	[122]
Synthetic compound				
	L002 and its analogs	p300,PCAF, GCN5	Suppress tumor growth and histone acetylation of breast cancer xenografts	[147]
	HATi II	p300, CBP	Induces cell cycle arrest and apoptosis in cancer cells	[146]
	C646	p300	Inhibits Ac-CoA and substrate binding. Suppress histone acetylation in human cells in vitro Inhibits cell proliferation and induces cell cycle arrest and apoptosis in cancer cells	[138–143]
Bisbenzamidine derivative	Pentamidine	Tip60	Inhibits acetylation of histone H2A, inhibits radiation-induced focus formation and homologous recombination repair pathway	[159]
Thiazole derivatives	CPTH2, CPTH6	GCN5/PCAF	Compete with substrates. Induce differentiation and apoptosis in cancer cells. Modulate autophagic flux in human cancer cells Selectively kill lung cancer stem cells	[152–155]
	CPTH derivatives	p300, PCAF	Induce apoptosis and cytodifferentiation in leukemia cells	[156]
	BF1	GCN5, p300	Competes with substrates. Induces histone hypoacetylation and apoptosis in human cancer cells	[151]
Isothiazolones derivatives	NU9056	Tip60	Is effective against prostate cancer cells	[158]
	TH1834	Tip60	Inhibits AcCoA binding. Sensitizes breast cancer cells to ionizing radiation	[161].
	PU141	KAT3	Exerts anti-proliferative properties on a panel of human cancer cell lines	[149]
	PU139	p300,CBP, PCAF, GCN5	Synergizes with doxorubicin and to reduces histone lysine acetylation in vivo at concentrations that block neoplastic xenograft growth	[149]
	5-Cloroisothiazolone	PCAF	not determined	[150]

## LIMITATIONS OF KAT INHIBITORS AND POTENTIAL ALTERNATIVE STRATEGIES

Almost all studies on KATi are currently still in a preclinical phase. Better agents, with greater specificity and better pharmokinetic properties are sorely needed. Nowadays, a compelling alternative to the direct targeting of KATs is coming forward based on the inhibition of BET family proteins, enzymes recognizing lysine acetylated residues [162, 163]. These inhibitors act as acetyl-lysine mimics that occupy the acetylated lysine-binding site in bromodomain-containing proteins. Pharmacological inhibition of BET proteins shows therapeutic activity in a variety of different pathologies, particularly in models of hematologic malignancies, cancer and inflammation [164–166]. Such effects have been attributed to a specific set of downstream target genes, whose expression is modulated by pharmacological targeting of BET proteins. Currently, there have been five registered active clinical trials investigating the targeting of BET family proteins, such as RVX-208, I-BET 762, OTX 015, CPI-0610 and TEN-010, which reported encouraging results in treating hematologic malignancies (https://clinicaltrials.gov/).

## CONCLUDING REMARKS AND FUTURE PERSPECTIVES

Lysine acetylation occurs not only at the histone tails but also in non-histone proteins. Importantly, aberrant lysine acetylation in cancer cells affects virtually all cellular pathways that have been associated to tumorigenesis. Furthermore, alterations in the function of histone-modifying complexes deregulate the control of chromatin-based processes, ultimately leading to oncogenic transformation and the development of cancer. Consistent with this notion, aberrant patterns of histone modifications and KATs mutations have been associated with a large number of human malignancies, and could provide cancer biomarkers for predicting prognosis and for determining better treatment options for cancer patients. Hence, the diverse role of KATs, the increasing numbers of KAT subtypes and the increasing evidence that the deregulation of epigenetic processes plays a key role in several diseases hold promise for future therapeutic strategies focused on KAT inhibition.

Extensive studies have explored small-molecule inhibitors of KAT family proteins for cancer therapy. However, they have not entered clinical trials. The non-specificity, pleiotropic effects, toxicity profiles and doses in the micromolar range are the major drawbacks in the KATi advancement. Curcumin, administered alone or with conventional drugs, is the only KATi tested in clinical trials against breast, prostate, colorectal and other solid cancers. However, it is important to note that curcumin shows enzymatic inhibitory activity on other proteins differently from KATs, which may explain the observed anticancer activity. Hence, the potential of using KATi for cancer therapy is still in its infancy.

A recent study carried out in Drosophila cells by Feller and collaborators [78], has elegantly demonstrated the role of different KATs in the acetylation of specific histone residues and highlighted as an increased knowledge of chromatin modification network is required for a more targeted utilization of epigenetic inhibitors in a clinical setting. Therefore, additional studies characterizing the mechanistic consequences of KATs overexpression or mutation in various cancers, as well as studies in animal models of disease, would be beneficial in understanding the role of several KATs in tumorigenesis. The identification of selective KATi would allow better design of combination therapies using these inhibitors in combination with other epigenetic agents, such as bromodomain inhibitors or conventional cancer therapy.
